# Research progress on the role and therapeutic applications of traditional Chinese medicine in radiation enteropathy

**DOI:** 10.3389/fphar.2026.1747735

**Published:** 2026-03-31

**Authors:** Yichao Liang, Hangping Chen, Guofei Ren, Guojun Jiang

**Affiliations:** 1 School of Pharmacy, Hangzhou Normal University, Hangzhou, Zhejiang, China; 2 Department of Pharmacy, Affiliated Xiaoshan Hospital, Hangzhou Normal University, Hangzhou, Zhejiang, China

**Keywords:** botanical medicine, clinical application, mechanism, radiation enteropathy, traditional Chinese medicine

## Abstract

Radiation enteropathy (RE) is a debilitating complication following radiotherapy, with currently limited treatment options. Multi-target intervention strategies such as Traditional Chinese Medicine (TCM) botanical formulas and acupuncture have been investigated as potential complementary approaches for its management. This review systematically synthesizes the evidence on TCM botanical formulas (e.g., Modified Baitouweng Decoction, Xihuang Pill, TJ-14) and acupuncture for the treatment of RE. Existing studies suggest multiple mechanisms of action, including anti-inflammatory and antioxidant effects, regulation of apoptosis and proliferation, restoration of intestinal barrier integrity, and modulation of gut microbiota. Clinical studies have reported improvements in symptoms and reductions in inflammatory markers, while preclinical models have demonstrated protective effects against radiation-induced intestinal injury. However, the current body of evidence is generally constrained by methodological limitations. Many clinical studies have small sample sizes and lack rigorous designs (e.g., absence of randomized controls), while mechanistic research often provides limited causal inference and relies on models with questionable clinical relevance, resulting in a significant translational gap. Based on this appraisal, we critically evaluate the limitations of existing research and propose future directions. These include: (i) employing CRISPR-based microbiome editing to investigate causal mechanisms; (ii) developing pharmacokinetic-pharmacodynamic (PK-PD) models for dose individualization; and (iii) utilizing innovative trial designs such as Bayesian adaptive trials to bridge the gap between empirical practice and evidence-based medicine. Rigorous further investigation is essential to define the role of these TCM interventions within the integrative treatment strategy for RE.

## 1 Radiation enteropathy (RE)

RE is a form of intestinal injury induced by radiotherapy, commonly observed in patients undergoing abdominal or pelvic radiation for malignant tumors. Its pathogenesis primarily stems from direct damage caused by ionizing radiation to intestinal tissues, leading to functional impairment and structural abnormalities (Ali and Radwan, 2021; Reis Ferreira et al., 2019). Pathologically, the disease involves intestinal epithelial cell injury, activation of inflammatory responses, and disruption of the intestinal microenvironment. Radiation can cause the depletion of intestinal stem cells, impair mucosal regeneration, and induce oxidative stress, thereby reducing the activity of antioxidant enzymes—such as glutathione peroxidase, superoxide dismutase, and catalase—while promoting the expression of pro-inflammatory cytokines, including (TNF-α), (IL-1β), (IFN-γ) (Moraitis et al., 2025).

This condition is particularly prevalent among patients receiving pelvic or abdominal radiotherapy for prostate cancer, gynecologic malignancies (e.g., cervical or endometrial cancer), and colorectal cancer. Studies indicate that approximately 25% of patients undergoing pelvic irradiation experience varying degrees of intestinal dysfunction, including radiation-induced enteritis, fibrosis, and luminal stenosis. Acute symptoms often include diarrhea and abdominal pain, whereas chronic manifestations may progress to intestinal fibrosis, stricture, or even fistula formation, significantly compromising patients’ quality of life and potentially necessitating surgical intervention with associated risks (Liu et al., 2023; Gnanapandithan et al., 2024).

The development of radiation enteropathy is closely linked to direct mucosal damage and subsequent gut microbiota dysbiosis, which can further perpetuate chronic inflammation and tissue fibrosis. The excessive generation of ROS induced by radiation also plays a pivotal role in intestinal injury (Yang et al., 2023). Given the multifactorial and multistage nature of its pathology, monotherapeutic approaches often fail to comprehensively manage the condition. Current pharmacological strategies primarily focus on anti-inflammatory and antioxidant mechanisms (Kwak et al., 2021; Jang et al., 2022). For instance, NADH has been shown to alleviate intestinal damage by suppressing inflammation and enhancing autophagy; however, its clinical application remains limited due to individual variability, low bioavailability, and concerns regarding long-term safety (Lu et al., 2025). In severe cases, surgical intervention may be required but carries risks of complications such as enteric fistula and infection, with some patients requiring multiple operations. Therefore, developing safe, effective, and sustainable alternative or adjunctive therapeutic strategies has become a research priority.

## 2 Traditional Chinese medicine (TCM)

Against this backdrop, the clinical management of RE continues to face challenges. Modern medicine primarily employs symptomatic treatments such as anti-inflammatory agents, anti-diarrheal drugs, and nutritional support. While these approaches can provide short-term symptom control, their effects are relatively limited in promoting the fundamental repair of the mucosal barrier, regulating long-term immune homeostasis, and restoring intestinal microbial ecology. Moreover, certain medications raise concerns regarding long-term safety. TCM, with its holistic philosophy and syndrome differentiation-based treatment system, offers an important complementary perspective. TCM interventions typically emphasize integrated regulation. Through multi-metabolite, multi-target, and multi-pathway actions, they aim not only to alleviate core symptoms such as diarrhea and abdominal pain but also to restore intestinal mucosal integrity, modulate immune balance, and correct dysbiosis, demonstrating potential for good long-term safety in management. Certainly, the application of TCM must also confront practical challenges, including the need for further clarification of its mechanisms of action, enhancement of the standardization of preparation quality control, and the urgent demand for large-scale, high-quality clinical research evidence. It is noteworthy that characteristic TCM therapies such as external treatments are gaining increasing attention due to their non-invasive nature and favorable safety profile. A systematic review and meta-analysis has shown that TCM external treatments can significantly improve the clinical symptoms of radiation enteropathy, providing preliminary evidence-based support for their clinical application (Luo et al., 2021).

According to TCM theory, RE can be attributed to “heat-toxin damaging yin” and “blood stasis obstructing the collaterals.” Radiation is viewed as a pathogenic “heat-toxin” that injures the intestinal collaterals and consumes qi and yin, leading to disruption in the circulation of qi and blood. This disruption triggers a cascade of pathological changes.

From a modern biomedical perspective, “heat-toxin” primarily refers to the acute injury process induced by radiation as an exogenous pathogenic factor, which closely correlates with the inflammatory cascade, oxidative stress burst, and immune activation recognized in modern medicine. “Damaging yin” encapsulates the progressive tissue damage and functional impairment caused by radiation. Its modern connotation encompasses the destruction of the intestinal mucosal barrier, microcirculatory disturbances, abnormally increased apoptosis, and fibrotic lesions resulting from imbalanced tissue repair in later stages. This understanding of the disease mechanism suggests that TCM’s approach to preventing and treating radiation enteropathy is not based on vague traditional experience but is grounded in a holistic grasp of its key pathological links—namely, the dynamic process from acute inflammatory and oxidative damage to chronic structural and functional abnormalities (Wu W. et al., 2025; Hu et al., 2025). TCM treatment principles focus on clearing heat and toxins, promoting blood circulation, resolving stasis, and strengthening the spleen and stomach to restore intestinal function and alleviate symptoms (Yan et al., 2023). For example, heat-clearing and detoxifying botanical drugs such as Coptis chinensis Franch. (Ranunculaceae) and Scutellaria baicalensis Georgi (Lamiaceae), as well as spleen-strengthening botanical drugs such as Atractylodes macrocephala Koidz. (Asteraceae) and Poria cocos (Schwein.) F.A. Wolf (Polyporaceae), are commonly used in the management of intestinal disorders (Luo et al., 2021).

## 3 Mechanisms of traditional Chinese medicine in treating radiation enteropathy

### 3.1 Anti-inflammatory and antioxidant effects

Preclinical studies have explored the potential of botanical metabolites to counteract oxidative stress and inflammation associated with radiation-induced intestinal injury. *In vitro* and animal model data suggest compounds such as flavonoids (e.g., baicalein, baicalin), terpenoids (e.g., paeoniflorin), and alkaloids may modulate these processes. For instance, baicalein has been reported to reduce leukocyte infiltration in irradiated rodents, and extracts of Gegenqinlian Decoction (GQD) (Refer to the Appendix) have shown downregulation of inflammatory cytokines in similar models, with mechanisms often attributed to inhibition of the NF-κB pathway (Jang et al., 2019; Zhang et al., 2026). Isolated compounds like paeoniflorin and glycyrrhizic acid from Shaoyao Gancao Decoction (SYD) (Refer to the Appendix) also demonstrate NF-κB inhibitory activity in cellular systems (Li Z. et al., 2022). Other reported effects include modulation of the TLR4/MyD88 axis by baicalin (Lu et al., 2023), enhancement of antioxidant enzymes by Astragalus polysaccharide (Lu et al., 2023; Zhang et al., 2023), and activation of the Nrf2 pathway by curcumin (Gao et al., 2024).

These preclinical studies provide preliminary evidence for the anti-inflammatory and antioxidant mechanisms of TCM in the treatment of RE. In particular, investigations into compound formulas such as GQD and SYD, as well as individual constituents like baicalein and paeoniflorin, suggest potential pathways for alleviating radiation-induced intestinal injury through the regulation of key signaling cascades, including NF-κB and Nrf2.

However, much of the key evidence is derived from simplified experimental systems. For instance, *in vitro* studies of isolated compounds frequently employ concentrations in the range of 10–100 μM, which may exceed achievable plasma or tissue concentrations *in vivo*, and their bioavailability and metabolic profiles remain poorly characterized (Wang K. et al., 2026; Kamiya et al., 2022). Moreover, the commonly used acute high-dose radiation animal models do not fully recapitulate the fractionated radiotherapy regimens and patient heterogeneity encountered in clinical practice (Zhao et al., 2023; Bloch et al., 2012). It should be emphasized that, due to ethical constraints, radiation injury studies cannot be conducted directly in humans; thus, animal models currently possess an indispensable scientific rationale and their value in preliminary mechanistic exploration merits recognition. Certain constituents (e.g., baicalein, curcumin) exhibit PAINS (pan-assay interference compounds) characteristics, and their apparent activity in routine assays may be subject to non-specific interference. This underscores the need for future studies to employ orthogonal experimental approaches to assess whether observed effects reflect specific target engagement or non-specific interference, rather than negating the potential value of these compounds.

### 3.2 Regulation of apoptosis and proliferation

Preclinical studies propose that select botanical interventions may modulate radiation-induced apoptosis and promote cellular regeneration in the intestine. *In vivo* reports indicate that formulas like Buzhong Yiqi Decoction (BZYQD) (Refer to the Appendix) can be associated with suppressed p53 signaling and reduced enterocyte apoptosis in irradiated animals. Similarly, GQD and certain isolated compounds, such as the saponin notoginsenoside R1, have been linked in experimental settings to the promotion of proliferation (via Wnt/β-catenin signaling) and inhibition of apoptosis (e.g., via Bax/caspase-3 pathway modulation), respectively (Zhang et al., 2026; Lu et al., 2023; Kang et al., 2023).

These findings suggest that formulas such as Buzhong Yiqi Decoction and Gegenqinlian Decoction, along with isolated compounds like notoginsenoside R1, may modulate apoptosis and proliferation through pathways including p53, Wnt/β-catenin, and caspase signaling. However, these observations remain at the hypothesis-generating stage.

However, the current body of evidence is largely derived from acute radiation models or homogeneous cell lines, which do not fully capture the chronic and multifactorial nature of clinical RE (Dejonckheere et al., 2026; Kamiya et al., 2015; Verginadis et al., 2025). Moreover, certain bioactive constituents—such as specific flavonoids and saponins—may generate signals *in vitro* that warrant further evaluation in physiologically relevant systems due to structural features associated with PAINS liabilities due to structural features associated with PAINS liabilities (Sheridan and Spelman, 2022). This does not negate the potential pharmacological value of these compounds, but rather highlights the need for orthogonal assays (e.g., target engagement studies, structure–activity relationship analysis) to assess whether observed effects arise from specific mechanisms or non-specific interference, thereby more precisely defining their therapeutic relevance.

Future research should prioritize evaluation in chronic or genetically diverse disease models, and employ deconvolution strategies alongside orthogonal detection methods to distinguish the specific contributions of individual components within complex formulas from non-specific effects.

### 3.3 Restoration of intestinal barrier function

Radiation injury compromises the intestinal barrier, increasing permeability and the risk of bacterial translocation. Preclinical studies propose that some botanical interventions may enhance barrier function, in part through the upregulation of tight junction and associated proteins. For example, Xuebijing Injection (XBJ) (Refer to the Appendix) has been reported in animal studies to upregulate tight junction protein expression, correlating with ameliorated barrier damage. Similarly, LGYD is described as promoting intestinal stem cell regeneration and crypt reconstruction in model systems (Yan et al., 2023; Dong et al., 2022; Yan et al., 2024). Furthermore, isolated compounds such as certain polyphenolic tannins (e.g., from pomegranate pericarp) have shown an ability to enhance the expression of occludin and claudin-1 *in vitro*, reducing epithelial leakage (Wu Z. et al., 2023).

Interventions such as XBJ and LGYD have been shown in animal models to enhance tight junction protein expression and promote intestinal stem cell regeneration, offering promising directions for repairing radiation-induced intestinal barrier damage.

The evidence supporting these conclusions is derived primarily from rodent models of acute total-body or abdominal irradiation, which differ from the focal, fractionated radiotherapy regimens encountered in clinical practice. The specific bioactive components responsible for these effects, as well as their achievable exposure levels within intestinal tissues, remain poorly characterized. Moreover, the *in vitro* “barrier-strengthening” effects observed for certain polyphenolic monomers require further investigation using orthogonal techniques to determine whether they result from specific modulation of tight junction dynamics or from non-specific protein interactions (consistent with PAINS-related characteristics). Such investigation is essential to clarify their pharmacological mode of action.

Future research should prioritize the development of barrier injury models that more closely recapitulate the clinical setting, with an emphasis on assessing long-term, functional outcomes of barrier integrity. For individual compounds, pharmacological relevance must be evaluated through rigorous target engagement studies to determine whether proposed mechanisms are operative under physiologically relevant conditions.

### 3.4 Modulation of gut microbiota

Radiotherapy is frequently associated with gut microbiota dysbiosis, marked by a decline in beneficial bacteria. Preclinical investigations have reported associations between certain botanical interventions and modifications in gut microbiota composition following radiation injury. For instance, administration of Liangxue Guyuan Yishen Decoction (LGYD) to irradiated mice has been correlated with an increased abundance of Akkermansia muciniphila and elevated levels of fecal short-chain fatty acids (SCFAs), with the latter proposed to support mucosal repair) (Yan et al., 2024). It is proposed that these SCFAs, such as isobutyrate, may then support epithelial repair and stem cell recovery, though this causal chain remains to be experimentally tested *in vivo*. Similarly, polysaccharide extracts from sources like Poria cocos are reported in experimental studies to promote beneficial bacteria and suppress pathogens (Lu et al., 2023; Zhang et al., 2023).

A critical appraisal reveals that this evidence remains preliminary and is constrained by significant methodological limitations that preclude definitive causal inferences. The primary data are derived from correlative observations in rodent models, typically utilizing 16S rRNA gene sequencing which lacks the resolution to identify functionally relevant bacterial strains. Crucially, these studies almost universally lack causal experimental designs, such as the use of germ-free animals or fecal microbiota transplantation, to demonstrate that the observed microbial shifts are necessary and sufficient for the purported therapeutic effects. Furthermore, the chemical complexity of the interventions presents a major challenge for reproducibility and mechanistic interpretation. For botanical decoctions like LGYD, the specific metabolites driving the microbial changes are undefined. For polysaccharide preparations, their high molecular weight and structural heterogeneity result in substantial batch-to-batch variability, a critical factor influencing prebiotic activity that is rarely quantified or reported in the cited studies. Therefore, while these findings suggest a plausible interaction between botanical drugs and the gut microbiome, they establish correlation, not causation. The hypothesis that microbiota modulation is a primary therapeutic mechanism remains to be experimentally tested. Future research must employ causal experimental models and pair microbial profiling with detailed chemical characterization of the interventions to bridge this evidence gap.

### 3.5 Multi-target synergistic effects

A central tenet in the study of complex botanical drugs is the proposed multi-target, synergistic action of their constituent metabolites. For instance, preclinical data propose that GQD exerts combined anti-inflammatory, anti-apoptotic, and pro-proliferative effects via the coordinated actions of compounds like puerarin, baicalein, berberine, and glycyrrhizic acid on pathways such as NF-κB, Caspase-9/-3, and Wnt/β-catenin (Zhang et al., 2026).

The concept of “multi-target synergy” represents a core and theoretically sound framework for explaining the holistic efficacy of TCM formulas. Studies on formulas such as Gegenqinlian Decoction suggest that their multiple constituents may act synergistically through pathways including NF-κB, Caspase, and Wnt, thereby exerting integrated anti-inflammatory, anti-apoptotic, and pro-proliferative effects.

At present, this hypothesis is largely inferred from phenotypic observations of whole-extract formulations. Certain constituents within these formulas—such as baicalein and berberine—possess structural features that warrant further evaluation (PAINS-associated alerts) (Li J. et al., 2022). Their activities observed in simplified experimental systems should be further investigated using orthogonal assays to assess target specificity.

Future research should employ systems pharmacology approaches to explore the interaction patterns of individual components at physiologically relevant ratios. Such efforts are essential to distinguish genuine synergistic effects (beyond additive effects) from simple additivity, and will contribute to a more mechanistic understanding of the holistic therapeutic mechanisms underlying complex formulas (Xu et al., 2021).

## 4 Mechanisms of action of traditional Chinese medicine therapies (acupuncture and massage)

### 4.1 Acupuncture

#### 4.1.1 Regulation of lymphatic circulation

Acupuncture modulates lymphatic system function by stimulating specific acupoints, thereby promoting lymph flow and aiding in the reduction of edema and inflammation. Studies have shown that acupuncture significantly enhances lymphatic drainage efficiency, particularly in radiation-affected areas. For instance, stimulation of the Houhai (HV) acupoint in animal models has been demonstrated to markedly improve lymphatic drainage and activate multiple lymph nodes, including the inferior mesenteric, inguinal, and internal iliac nodes. This effect may be attributed to acupuncture-induced local vasodilation and increased frequency of lymphatic vessel contractions (Jin et al., 2020).

#### 4.1.2 Modulation of inflammation and immune response

Radiation enteropathy is often accompanied by significant inflammation and immune dysregulation. Acupuncture can effectively suppress excessive inflammation by modulating the distribution and function of immune cells within the lymphatic system. For example, it reduces the proportion of M1 macrophages and decreases the numbers of monocytes and neutrophils, thereby mitigating immune overactivation (Wang J. et al., 2024). Additionally, acupuncture enhances the function of lymphatic endothelial cells and facilitates the clearance of inflammatory cytokines (Schwartz et al., 2019). It also activates immune cells and promotes the expression of cytokines such as IL-2, IL-4, and IFN-γ, thereby enhancing anti-inflammatory responses and tissue repair (Jin et al., 2020). Through its regulatory effects on the neuroendocrine system and inflammation, acupuncture may also help alleviate associated pain.

#### 4.1.3 Promotion of intestinal functional recovery

Effective repair mechanisms are essential for recovering from tissue damage caused by radiation enteropathy. Acupuncture promotes neural and vascular regeneration, accelerating tissue restoration. Studies indicate that acupuncture can repair spinal astrocytes and neuronal structures, improve the reinnervation rate of neuromuscular junctions, and thereby enhance tissue functionality. Furthermore, it facilitates lymphatic vessel regeneration and functional recovery, further accelerating the repair process (Wang J. et al., 2024).

Notably, acupuncture trials reviewed lacked sham-controlled double-blinding, making it impossible to exclude placebo effects or enhanced care effects (non-specific efficacy contributors).

The evidence supporting acupuncture’s mechanisms, while suggestive, is constrained by significant methodological limitations. Many cited studies employ animal models whose translational relevance to human pathophysiology remains uncertain. The noted absence of rigorous sham-controlled, double-blinded trials in clinical research is a critical shortcoming, as it prevents the differentiation of specific neurophysiological effects from potent placebo responses or the contextual effects of therapeutic attention. Furthermore, mechanistic explanations often rely on correlative observations (e.g., changes in cytokine levels or lymphatic flow post-stimulation) rather than experiments that definitively establish causal links between acupoint stimulation and the observed biological outcomes. Future research must prioritize high-quality clinical trials with robust blinding and credible sham controls, alongside mechanistic studies designed to isolate and verify causality.

### 4.2 Massage therapy

#### 4.2.1 Enhancement of local blood circulation

The pathogenesis of radiation enteropathy is closely linked to persistent damage and impaired regeneration of the intestinal mucosa following radiotherapy. Studies suggest that symmetric division of intestinal crypts plays a critical role in mucosal regeneration, a process tightly regulated by the WNT4 signaling pathway. Massage therapy may improve local blood circulation, enhance microvascular perfusion and oxygen supply, and thereby accelerate the repair of damaged mucosa and reduce inflammatory responses (Cheng et al., 2024).

#### 4.2.2 Improvement of lymphatic drainage

Through specific techniques and mechanical stimulation, massage promotes lymph flow and facilitates the clearance of metabolic waste and inflammatory factors (Palmer, 2024; Ramadan, 2024). Research has shown that massage increases the frequency and intensity of lymphatic vessel contractions, improving lymphatic function and effectively reducing tissue edema and local inflammation (Pawar et al., 2022). In radiation enteropathy, radiotherapy often impairs intestinal lymphatic drainage. Massage aids in alleviating intestinal edema and controlling inflammatory responses by promoting regional lymphatic circulation (Dong et al., 2024; De Vrieze et al., 2022).

**FIGURE 1:**
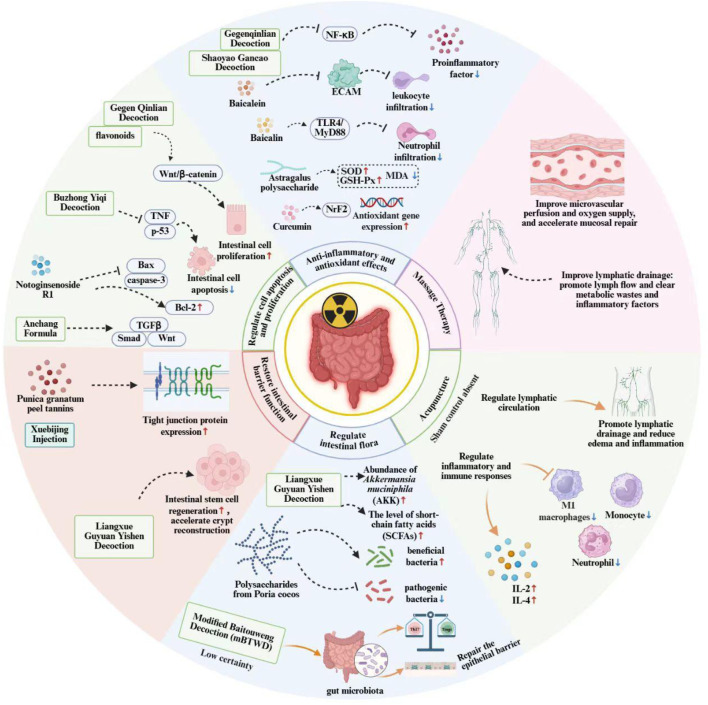
Schematic representation of hypothesized multi-target mechanisms for selected TCM interventions in RE (Schematic created by the authors).

The theoretical mechanisms proposed for massage therapy are plausible but require more robust empirical substantiation. Current evidence is largely preclinical or derived from small-scale, uncontrolled clinical observations. A major challenge is the inherent difficulty in standardizing massage interventions (e.g., pressure, technique, duration) and creating credible sham controls for blinding, which complicates the objective evaluation of its specific efficacy. Claims regarding the modulation of specific signaling pathways (e.g., WNT4) are primarily extrapolated from general physiological knowledge rather than direct evidence from massage studies in the context of radiation injury. To advance this field, studies must employ standardized protocols, develop methodologically sound sham techniques, and integrate objective biomarkers to distinguish true physiological effects from general relaxation responses (Figure 1).

Interpretation and key caveats:

1. Hypothesis summary: This diagram synthesizes mechanistic pathways proposed primarily by preclinical studies and, in some cases, preliminary clinical observations. It illustrates a network of hypotheses regarding how certain TCM formulas and external therapies might modulate key pathological processes in RE.
2. Evidence gradient: The depicted effects and pathways are not equally validated. They exist on an evidence continuum:

Some pathways (e.g., anti-inflammatory effects of certain formulas) are supported by clinical trial data, though often from studies with methodological limitations.

Many pathways (e.g., specific signaling modulation by isolated metabolites like baicalein) are derived from cell-based or animal models, and their clinical translatability remains to be confirmed.

Several components (e.g., metabolites flagged as pan-assay interference compounds, PAINS) have mechanistic claims based on *in vitro* data that require cautious interpretation and *in vivo* evaluation.

3. Not a panacea: This schematic does not imply universal or guaranteed efficacy of TCM for RE. Therapeutic outcomes are variable and depend on numerous factors including disease stage, individual patient biology, and treatment regimen. The “multi-target” nature depicted is a proposed model, not an established guarantee of clinical success.
4. Purpose: The figure serves to visually organize the complex and fragmented mechanistic literature discussed in this review, thereby highlighting both potential therapeutic strategies and the significant translational gaps that necessitate future rigorous research.

## 5 Application of traditional Chinese medicine in radiation-induced enteritis

### 5.1 Shaoyao decoction (SYD)

SYD (Refer to the Appendix), a classical botanical formula, is traditionally used for digestive disorders, with contemporary research exploring its potential application in radiation enteropathy. Preclinical investigations, primarily in mouse models, report that this multi-herb formulation can alleviate symptoms of radiation-induced enteritis, with proposed mechanisms involving the reduction of inflammation, inhibition of apoptosis, and amelioration of tissue fibrosis (Li Z. et al., 2022) Similar therapeutic effects have been observed in rodent studies of other formulas, such as Tongxie Formula and Modified Xijiao Dihuang Decoction (Yang et al., 2020; Wang et al., 2021).

Preclinical studies on classical formulas such as SYD have provided preliminary evidence of anti-inflammatory effects in animal models, warranting further investigation.

However, this body of evidence is derived predominantly from acute, high-dose radiation models in genetically homogeneous mice, which differ substantially from clinical radiotherapy regimens. Most studies have employed empirically fixed doses of whole aqueous extracts, lacking systematic dose–response characterization, pharmacokinetic profiling, or positive controls aligned with standard clinical care. The chemical complexity of the whole formula further obscures the contributions of individual bioactive constituents.

To facilitate clinical translation, rigorous trials incorporating pharmacokinetic–pharmacodynamic (PK–PD) modeling are urgently needed to establish rational dosing regimens and to distinguish specific pharmacological effects from non-specific mixture-related activities.

### 5.2 Modified Baitouweng Decoction (mBTWD)

BTWD (Refer to the Appendix), a traditional formula used for intestinal ailments, has been investigated in modern preclinical studies for its potential multi-target effects, including modulation of autophagy, oxidative stress, and immune balance (Zhang et al., 2024; Pan et al., 2024; Wei et al., 2022; Miao et al., 2020). A meta-analysis of clinical studies appears to support its utility, reporting that a mBTWD improved gastrointestinal symptoms, reduced inflammatory markers like CRP, and enhanced performance status in patients with radiation enteropathy across 17 included trials (Wu Z. et al., 2023).

A meta-analysis of clinical studies on Modified Baitouweng Decoction, encompassing 17 trials, suggests that this intervention may improve symptoms and reduce inflammatory markers in patients with RE, providing preliminary aggregated evidence to support its clinical application.

However, the original studies included in this meta-analysis exhibit substantial heterogeneity, with inconsistencies in formula composition, dosage, control interventions, and treatment duration. Most of these primary studies had small sample sizes and demonstrated inadequacies in the implementation and reporting of randomization and blinding methods, resulting in an overall risk of bias and limited generalizability of the evidence.

This positive signal warrants further evaluation through large-scale, multicenter randomized controlled trials employing standardized, quality-controlled interventions and rigorous double-blind, placebo-controlled designs.

### 5.3 TJ-14

TJ-14 (Refer to the Appendix) is a traditional botanical formulation used for gastrointestinal inflammation. Its potential application in acute radiation enteritis (ARE) was explored in a single-arm, multicenter Phase II trial (Yamashita et al., 2015; Chan et al., 2023; Murai et al., 2020). In this uncontrolled study of 22 patients, TJ-14 treatment was associated with a reduction in daily bowel frequency in 86% of participants, and the therapy was reported to be well-tolerated.

This Phase II single-arm trial of TJ-14 for the treatment of ARE has provided preliminary clinical data, suggesting a potential reduction in daily bowel frequency and favorable tolerability, thereby supporting further investigation of this formulation.

However, due to the non-randomized, uncontrolled single-arm design of this study, the observed symptomatic improvements cannot be distinguished from placebo effects or the natural fluctuation of disease symptoms (Bschor et al., 2024). The small sample size (n = 22) further limits the robustness and generalizability of the conclusions.

These encouraging results strongly support the initiation of large-scale, randomized, double-blind, placebo-controlled Phase III clinical trials to definitively evaluate the efficacy and safety of TJ-14.

### 5.4 Xihuang Pill (XHW)

XHW is a traditional botanical drug with reported antitumor and anti-inflammatory activities in oncology settings (Hong et al., 2014). To date, no empirical studies have investigated its application in RE. A network pharmacology analysis by Lv et al. (2024) proposed that certain XHW constituents—including quercetin, ellagic acid, and stigmasterol—might theoretically interact with pathways relevant to intestinal injury.

However, a recent large-scale methodological study by Diao et al. (2026) Diao et al., which systematically analyzed 1,038 network-based ethnopharmacology studies, has fundamentally challenged the interpretability of such computational predictions. This work demonstrates that the field suffers from a pervasive “homogeneity bias,” wherein a narrow set of molecules—most notably flavonoids like quercetin—are recurrently identified as “key metabolites” across disparate natural products and diseases. Crucially, this recurrence is not a reflection of genuine biological relevance, but rather an artifact of a self-reinforcing “convergent discovery pipeline.” This pipeline is characterized by: (i) initial biases within public databases; (ii) context-insensitive analytical algorithms; and (iii) the PAINS properties of these frequently flagged compounds, which generate spurious signals in conventional *in silico* assays.

Consequently, the predicted metabolites for XHW, such as quercetin and ellagic acid, are best understood as “chemical ghosts”—entities abundant in database entries but lacking evidence of *in vivo* bioavailability or meaningful systemic exposure following administration of the corresponding TCM formula. Their apparent “multi-target activity” *in silico* is, as Diao et al. argue, more likely to stem from these systemic methodological flaws than from specific, pharmacologically relevant target engagement. Therefore, the findings from Lv et al. cannot be considered predictive evidence of any kind. They should be regarded strictly as a hypothesis-generating exercise, the outputs of which are speculative and require rigorous empirical evaluation.

Future research on XHW for RE must therefore abandon reliance on purely computational predictions and prioritize empirical, data-driven approaches (i) systematic pharmacokinetic profiling (e.g., UHPLC-MS/MS): to identify constituents that are truly absorbed and reach intestinal tissue; (ii) bioactivity-guided fractionation to isolate and test active components; and (iii) target engagement studies using orthogonal assays (e.g., surface plasmon resonance, cellular thermal shift assays) to confirm specific metabolites. Until such empirical data are available, any proposed role for XHW in RE management remains entirely speculative and without evidentiary support.

### 5.5 Acupuncture combination therapy

Acupuncture is a component of traditional Chinese medicine with a long history of clinical use. It has been studied in a range of conditions, including certain endocrine, neurological, and musculoskeletal disorders (Wen et al., 2020; Wang et al., 2022). Research has also explored its potential role in managing gastrointestinal conditions, including inflammatory bowel disease (Li X. et al., 2022). In the specific context of radiation-induced enteritis, a meta-analysis by Wu et al. suggested that a combination of acupuncture and medication might lead to greater symptomatic improvement compared to medication alone, based on a synthesis of nine clinical studies (Li X. et al., 2022).

This meta-analysis suggests that combining acupuncture with pharmacotherapy may confer additional benefits in alleviating RE symptoms, supporting further exploration of integrative treatment strategies.

The original trials included in this analysis commonly face methodological challenges, including difficulties in implementing rigorous blinding (particularly practitioner blinding) and establishing credible sham acupuncture controls, which elevate the risks of performance bias and detection bias (Wang et al., 2022). Variability in acupuncture protocols—such as point selection and manipulation techniques—across studies further compromises the comparability of results.

To clarify the specific therapeutic contribution of acupuncture in RE management, future research should prioritize high-quality clinical trials with standardized intervention protocols, rigorous blinding designs, and credible controls.

The levels of evidence and mechanisms of action of various Traditional Chinese medicine interventions are summarized in Table 1.

**TABLE 1:** Summary of evidence for traditional Chinese medicine interventions in radiation enteropathy.

Intervention measure	Key active metabolites	Main mechanisms of action	Key experimental findings	Level of evidence	References
Shaoyao Decoction	Paeoniflorin, Oxypaeoniflorin, etc.	Anti-inflammatory, anti-apoptotic, amelioration of tissue fibrosis	Significantly alleviated symptoms of radiation-induced enteritis in C57BL/6 mice	Preclinical mouse model	Li Z. et al. (2022), Shou et al. (2018), Sun et al. (2022)
Modified Baitouweng Decoction	Anemoside B4,Berberine,β-peltatin	Anti-inflammatory, antioxidant, regulation of Th17/Treg balance, enhancement of intestinal barrier	Across 17 studies, showed symptom improvement, reduction in inflammatory markers, and increased KPS scores	Meta-analysis (clinical studies)	Wu Z. et al. (2023), Wu R. et al. (2023), Bai et al. (2025), Hu et al. (2022), Qiu et al. (2025)
TJ-14	Baicalein, licochalcone A,quercetin, naringenin	Anti-inflammatory, anti-oxidation, regulation of metabolism	In a Phase II trial, 86% of patients showed symptom improvement, with significant reduction in bowel frequency	Phase II clinical trial (single-arm)	Murai et al. (2020), Li C. et al. (2023), Yu et al. (2020), Shi Y. et al. (2024)
Xihuang Pill	Quercetin, ellagic acid, stigmasterol, etc. (predicted)	Antitumor, anti-inflammatory, modulation of PI3K-Akt, TNF, HIF-1 pathways	Network pharmacology Hypothesis-generating, potential use in radiation enteritis by targeting genes such as PTGS1, IL-6, CASP3	Hypothesis-generating only (awaiting experimental evaluation)	Lv et al. (2024)
Acupuncture Combination Therapy	Not applicable	Regulation of lymphatic circulation, suppression of inflammation, promotion of intestinal functional recovery	Acupuncture combined with medication was superior to medication alone in alleviating symptoms	Meta-analysis (clinical studies)	Li X. et al. (2022), Joung et al. (2024), Shi F. et al. (2024b)
Shaoyao Gancao Decoction	Paeoniflorin, glycyrrhizic acid	Inhibition of NF-κB pathway, reduction of IL-1β, IL-6, TNF-α	Reduced radiation-induced mucosal edema and inflammatory infiltration	Preclinical study	Zhu et al. (2024), Wu et al. (2024), Luo et al. (2023), Qin et al. (2023)
Liangxue Guyuan Yishen Decoction	Not specified	Promotion of intestinal stem cell regeneration, enhancement of barrier integrity, modulation of gut microbiota (e.g., Akkermansia)	Promoted intestinal stem cell regeneration and increased levels of short-chain fatty acids	Preclinical study	Yan et al. (2023), Yan et al. (2024), Yan et al. (2025)
Xuebijing Injection	Monoterpenoids	Anti-inflammatory, immunomodulatory, antioxidant properties	Significantly ameliorated radiation-induced intestinal barrier injury	Preclinical study	Dong et al. (2022), Li et al. (2020a)

Table 1 summarizes key TCM interventions for RE, detailing their active metabolites, mechanisms, findings, and evidence levels—categorized as preclinical models, clinical trials (Phases II/III), and meta-analyses to illustrate the translational pathway. Terminology aligns with the main text. Notably, some mechanisms (e.g., Xihuang Pill) rely on network pharmacology predictions awaiting experimental evaluation, and certain clinical studies (e.g., TJ-14) are single-arm trials requiring further assessment by robust RCTs. The synthesized information supports the discussion in Section 6 on evidence heterogeneity, methodological limitations, and translational challenges.

## 6 Discussion

### 6.1 Comparison of clinical findings and preclinical studies: addressing the translational gap

The current evidence system for botanical drugs in the treatment of radiation enteropathy exhibits a distinct “two-tiered structure”: a foundation of animal experiments with relatively clear mechanisms but simplified models, and an upper layer of clinical observations that suggest efficacy yet are marked by high heterogeneity and weak causal inference. A systematic comparison of these two tiers of evidence is instrumental in clarifying the central focus of translational research (Table 2).

**TABLE 2:** Comparative analysis of preclinical and clinical evidence.

Dimension of comparison	Key findings from preclinical animal studies	Key findings from clinical studies	Gaps and challenges
Efficacy endpoint	Focuses on surrogate endpoints at the molecular, histopathological, and barrier function levels, such as decline in inflammatory markers, increased expression of tight junction proteins, and improvement in gut microbiota α-diversity	Focuses on patient-reported symptom improvements (such as diarrhea frequency, abdominal pain relief), quality of life scores, and certain inflammatory markers (e.g., CRP)	There is a lack of bridging biomarkers that quantitatively link the microscopic improvements observed in animal models to clinically meaningful, patient-perceivable symptom relief
Mechanism interpretation	It is capable of mapping out the signaling pathways (e.g., NF-κB, Wnt/β-catenin, TLR4/MyD88, etc.) through which specific metabolites or metabolite formulations exert their effects, and can further establish causal relationships using techniques such as knockout models or specific inhibitors	These studies often remain at the level of describing phenomenological correlations (e.g., concurrent improvement in symptoms and microbiota changes following treatment), making it difficult to pinpoint the specific necessary and sufficient conditions for efficacy—such as which botanical metabolites, which bacterial strain, or which precise pathway is responsible	There exists a significant gap in simplification and evaluation between the “multi-target, multi-pathway” mechanistic map and the clinically verifiable and intervenable “key targets.”
Intervention fidelity	Experimental conditions are strictly controlled, with drug dosage, combination, and dosing regimens being highly standardized	There is considerable heterogeneity in interventions (e.g., modifications of botanical formulations, variations in acupuncture techniques), accompanied by a lack of standardized manufacturing quality control and systematic dose-finding studies	The optimal dosage and formulation identified in animal studies are difficult to translate directly and equivalently into highly individualized clinical practice
Model and population	The studies typically employ genetically homogeneous, healthy young animals subjected to standardized radiation-induced injury, resulting in a relatively homogeneous disease model	studies typically employ genetically homogeneous, healthy young animals subjected to standardized radiation-induced injury, resulting in a relatively homogeneous disease model. In contrast, patient populations exhibit substantial heterogeneity in terms of age, comorbidities, radiotherapy regimens, combination therapies, and baseline gut microbiota	Therapeutic strategies validated in homogeneous models may see their efficacy diluted or restricted to specific subgroups in heterogeneous patient populations, while current “one-size-fits-all” trial designs are ill-suited to identify such subgroups

Table 2 presents a systematic, four-dimensional comparison (efficacy endpoints, mechanism interpretation, intervention fidelity, and model/population) between preclinical and clinical evidence on TCM for RE. By juxtaposing the distinct methodologies, evidence levels, and generalizability across these two research tiers, it visually delineates the prevailing “translational gap.” This comparative framework underpins the subsequent critical discussion on methodological limitations (Section 6.2) and the formulation of proposed future research directions (Section 7).

In summary, preclinical studies provide a wealth of mechanistic hypotheses and proof-of-principle for efficacy, while clinical research offers preliminary human safety data and signals of therapeutic benefit. However, a distinct “translational gap” exists between the two: the former fails to adequately simulate the complexity of the clinical setting, while the latter often lacks the rigorous design necessary to validate the mechanistic hypotheses. This disconnect highlights the need to strengthen the evidence base for botanical drugs by developing more rigorous prospective research strategies that build upon existing mechanistic insights. Therefore, future research urgently needs to adopt translational strategies capable of bridging microscopic mechanisms with macroscopic phenotypes.

### 6.2 Systematic summary of methodological limitations

Although existing studies provide promising preliminary evidence for the use of botanical drugs and acupuncture in the treatment of radiation enteropathy, it must be clearly recognized that this evidence is generally constrained by significant methodological limitations, which compromise the reliability of its conclusions and their translation into clinical practice.

#### 6.2.1 Study design level

Lack of Rigorous Controls and Blinding: Most clinical reports are case series or single-arm trials, lacking randomization, placebo controls, or standard treatment controls. Even in studies with control groups, the implementation and reporting of allocation concealment and blinding (especially for practitioners and outcome assessors) are often inadequate, making it difficult to rule out placebo effects and observer bias.

Small Sample Sizes and Limited Representativeness: Existing clinical trials generally have small sample sizes (e.g., only 22 treated patients in the TJ-14 study), resulting in insufficient statistical power. Furthermore, study subjects are often recruited from single centers with relatively homogeneous populations, limiting the generalizability of findings to broader, more heterogeneous radiotherapy patient populations.

#### 6.2.2 Intervention and implementation level

High Heterogeneity and Insufficient Standardization: Considerable variability exists across studies regarding the specific composition, geographical origin of medicinal materials, dosage, preparation methods, and treatment duration of botanical drug formulas. Similarly, acupuncture studies lack standardization in point selection, manipulation techniques, stimulation parameters, and treatment frequency. This high degree of heterogeneity makes it difficult to compare, pool, and replicate study results.

Unclear Dose-Response Relationships: The conversion of doses from animal experiments to human trials often relies on traditional empirical ratios, lacking precision based on pharmacokinetic (PK) modeling. Human exposure levels of active metabolites, the therapeutic window, and dose-response curves remain unclear.

#### 6.2.3 Mechanistic research level

Weak Causal Inference: Gut microbiota research predominantly remains at the level of correlational description (e.g., 16S rRNA sequencing). Strategies such as using germ-free animals, fecal microbiota transplantation, or specific bacterial colonization to establish a causal chain from “microbiota alteration” to “functional improvement” are rarely employed. Network Pharmacology analyses should be regarded as hypothesis-generating tools only. Their outputs are prone to systematic biases—such as the recurrent identification of PAINS compounds—and cannot be interpreted as predictive of pharmacological activity without rigorous, multi-layered empirical evaluation.

##### 6.2.3.1 Model limitations

Preclinical research primarily relies on rodent models, which cannot fully replicate the complex pathophysiology and long-term chronic progression of human radiation enteropathy. Many *in vivo* studies lack standardized positive controls.

#### 6.2.4 Outcome assessment and reporting level

Lack of Uniform Outcome Measures: Inconsistencies exist across studies in the symptom assessment scales, biological markers, and measurement timepoints used, hindering comprehensive evaluation of the results.

Insufficient Systematic Safety Reporting: For complex multi-botanical formulas or long-term treatments, studies often lack systematic monitoring and reporting of adverse events. Particular attention is needed regarding potential hepatorenal toxicity, interactions with radiotherapy/chemotherapy, and long-term effects on the microbiome.

### 6.3 Evidence quality appraisal

To make the confidence in each finding explicit, we applied a modified GRADE framework to the key studies retrieved (Table 3). Randomized controlled trials (RCTs) began at “high” certainty and were down-graded for: (i) risk of bias (−1 if allocation concealment or double-blinding was inadequate); (ii) imprecision (−1 if the aggregate sample size was <50 patients per arm); (iii) inconsistency (−1 if I^2^ > 50% or point estimates were clinically disparate); and (iv) indirectness (−1 if botanical composition or outcomes differed materially from current clinical practice). Meta-analyses inherited the lowest certainty among their source trials and were penalised an additional level for unexplained heterogeneity. Single-arm phase II studies started at “low” certainty and were further down-graded for absence of control (−1) and small sample (−1). Pre-clinical experiments were defaulted to “very low” certainty; they could be up-graded only when at least two independent laboratories had replicated the effect and a dose–response relationship was demonstrated (Table 3).

**TABLE 3:** Evidence quality appraisal.

Intervention	Study type	No. of studies (participants)	Major limitations	PAINS liability	GRADE certainty
mBTWD	Meta-analysis of RCTs	17 (n = 1,611)	High heterogeneity (I^2^ > 50%), unclear randomization/blinding in original trials, botanical composition variations across studies	Medium (Contains alkaloids like berberine [potentially specific] but also flavonoids/tannins [PAINS-prone]; extraction methods inconsistently reported)	Low
TJ-14	Phase II trial	1 (n = 22)	Single-arm, no placebo control, small sample, selection bias possible	High (Flavonoid-rich Scutellaria and Glycyrrhiza extract; high content of baicalin/glycyrrhizin with established PAINS filters)	Very low
Xihuang pill	Network pharmacology	1 *in silico* study	Predictive only; awaiting experimental evaluation; predicted metabolites (quercetin, ellagic acid) are implausible due to homogeneity bias and PAINS properties; algorithms cannot distinguish specific binding from non-specific interference	Critical (Quercetin, ellagic acid, stigmasterol are archetypal PAINS: catechol, polyphenol, and steroid scaffolds known for assay interference)	Very low
Acupuncture combination	Meta-analysis	9 RCTs (n = 704)	Sham control absent in all studies, high clinical heterogeneity (acupoint selection, stimulation parameters), practitioner blinding impossible	N/A (Non-pharmacological intervention)	Low
Shaoyao decoction	Preclinica	Multiple mouse/rat studies	No standardized dosing, lack of positive controls, limited replication across labs	High (Dominant flavonoids [e.g., paeoniflorin/glycyrrhizin] contain PAINS substructures; claims of NF-κB inhibition based on *in vitro* assays prone to artifact)	Very low
Gegenqinlian/Shaoyaogancao Decoctions	Mechanism studies	*In vitro*/cellular	No orthogonal evaluation (e.g., SPR/DLS for aggregation), micromolar IC50 values in buffered aqueous systems (unphysiological)	Critical (Multiple flavonoid glycosides with catechol and quinone PAINS alerts; redox cycling likely confounds ROS readouts)	Very low

PAINS assessment based on structural filters per Bolz et al. (2021) and Baell and Walters (2014). Critical = >2 PAINS filters present; High = 1-2 PAINS filters; Medium = potential for interference depending on formulation; Low = specific alkaloids with defined targets.

As summarised in Table 3, the body of evidence supporting mBTWD for symptomatic relief is rated as low certainty (serious risk of bias and marked heterogeneity), whereas the single-arm TJ-14 trial and the network-pharmacology analysis of XHW are both classified as very low certainty. Consequently, this review does not generate actionable clinical recommendations; rather, it delineates the evidence landscape and highlights the urgent need for large-scale, rigorously controlled trials.

### 6.4 The PAINS problem: pharmacological credibility and evidence re-evaluation of reported mechanisms

A critical methodological limitation prevalent in the current mechanistic literature on TCM for radiation enteropathy is the insufficient scrutiny and exclusion of PAINS. PAINS are chemically promiscuous molecules whose apparent “bioactivity” often stems from interference with the assay system itself, rather than genuine modulation of specific biological targets—a well-recognized pitfall in modern drug discovery (Bolz et al., 2021; Baell and Walters, 2014; Magalhães et al., 2021).

Attention to the issue of PAINS reflects a growing demand for higher quality in pharmacological research on TCM. It contributes to enhanced rigor and reproducibility in mechanistic studies and represents an important step in advancing the modernization of TCM.

Many mechanistic studies in this field do indeed involve compounds with PAINS-alerting structural features (e.g., flavonoids, polyphenols, and certain terpenoids). When conclusions regarding specific target engagement are drawn solely from conventional *in vitro* assays—such as NF-κB reporter gene assays or antioxidant activity tests—the strength of such evidence may be insufficient, as the observed activities may arise from non-specific interference.

We recommend that future mechanistic studies incorporate PAINS awareness at the design stage. For compounds with PAINS alerts, orthogonal experimental evidence (e.g., surface plasmon resonance, dynamic light scattering, cellular thermal shift assays) should be provided to assess whether observed activities arise from specific target engagement or assay interference, and activity should be reported at physiologically relevant concentrations. Such approaches will substantially enhance the credibility and translational potential of research findings in this field (Ardenkjær-Skinnerup et al., 2023; Fotsch et al., 2024; Rothenaigner and Hadian, 2021).

## 7 Limitations and future research directions

Based on the aforementioned methodological limitations, future research must focus on the following directions to build a more robust and translatable evidence base:

Conduct rigorously designed randomized controlled trials (RCTs): Priority should be given to advancing large-scale, multicenter, randomized, double-blind, placebo-controlled Phase III clinical trials. Trial designs should clearly define primary and secondary endpoints, employ recognized assessment tools, and strictly implement blinding and allocation concealment. For acupuncture studies, appropriate “sham acupuncture” or simulated treatments should be explored and employed as controls.

### 7.1 Investigating mechanisms and establishing causality

Utilize germ-free animals, human microbiota-colonized mice, and gene-editing technologies such as CRISPR to directly test the causal pathway by which specific botanical drug metabolites improve the intestinal barrier and symptoms through the regulation of key bacterial strains and their metabolites (e.g., short-chain fatty acids).

Employ *in vitro* models like organoids and organ-on-a-chip systems, combined with molecular biology techniques, to precisely dissect the targets and signaling networks of bioactive botanical drug metabolites at the cellular and pathway levels.

### 7.2 Advancing pharmacokinetic-pharmacodynamic (PK-PD) integration and dose optimization research

Systematically conduct PK studies of botanical drug formulas and their active metabolites in healthy volunteers and patients to clarify their absorption, distribution, metabolism, and excretion profiles.

Develop integrated mathematical models that combine “microbiome signatures - host metabolic phenotypes - drug PK parameters - clinical PD indicators” to enable a shift from crude weight-based dosing (g·kg^−1^) to precise dosing based on target exposure or local intestinal concentration (e.g., μM·g^−1^ of intestinal content).

### 7.3 Developing stratified and personalized treatment strategies

Prospectively collect patient baseline characteristics (e.g., microbiome profiles, inflammatory markers, radiation field and dose) in clinical trials, and use methods like machine learning to identify biosubtypes that respond well to specific botanical drug therapies.

Explore adaptive clinical trial designs (e.g., Bayesian platform trials) that allow for dynamic adjustment of groupings to efficiently evaluate efficacy within specific subpopulations.

### 7.4 Strengthening research on long-term safety and interactions

In long-term follow-up clinical trials, systematically monitor the effects of botanical drug treatments on liver and kidney function, coagulation profiles, and the resistome of the gut microbiota.

Investigate the pharmacokinetic and pharmacodynamic interactions between botanical drugs commonly used for radiation enteropathy and chemotherapy or targeted therapies.

### 7.5 Promoting methodological standardization and reporting norms

Promote the development of a core outcome set (COS) and standardized intervention reporting guidelines specific to botanical drug therapy for radiation enteropathy.

Encourage researchers to adhere to international norms such as the CONSORT statement (and its extensions for botanical interventions) and the STRICTA guidelines for reporting acupuncture trials in their research and publications.

By focusing on the above research directions, it is anticipated that the current challenges of fragmented evidence and low translatability can be overcome. This will facilitate the evolution of botanical drug therapy for radiation enteropathy from an empirical practice towards an evidence-based model characterized by clear causal logic, precise dosing, and well-defined patient populations who stand to benefit.

## 8 Conclusion

This review synthesizes recent evidence to systematically elucidate the mechanisms and efficacy of TCM and acupuncture in alleviating radiation enteropathy through multi-target networks. It highlights translational challenges such as fragmented causality, lack of dose conversion, and unstratified populations. Furthermore, it proposes a precision evidence-based framework centered on CRISPR-assisted microbiota editing, PK-PD bridging, and Bayesian adaptive design, providing a methodological roadmap for advancing TCM into international Phase III trials and inclusion in RE treatment guidelines.

## Appendix

**Table:**

Botanical formula	Composition	Key active metabolites	Pharmacological effects	References
Gegenqinlian Decoction	*Pueraria montana* var. *lobata* (Willd.) Maesen and S.M.Almeida ex Sanjappa and Predeep [Fabaceae], *Scutellaria baicalensis* Georgi [Lamiaceae], *Coptis chinensis* Franch. [Ranunculaceae], *Glycyrrhiza uralensis* Fisch. ex DC. [Fabaceae]	Puerarin, Baicalin, Berberine, Glycyrrhizic acid	Anti-inflammatory effects, inhibition of apoptosis, and promotion of intestinal epithelial repair and regeneration	Bolz et al. (2021), Li et al. (2021), Song et al. (2022)
Shaoyao Gancao Decoction	*Paeonia lactiflora* Pall. [Paeoniaceae], *Glycyrrhiza glabra* L. [Fabaceae]	Paeoniflorin, Glycyrrhizic acid	Anti-inflammatory action, modulation of gut microbiota, and protection of the intestinal barrier	Hong and Xiao (2025), Li et al. (2025), Facheng et al. (2025), Wu L. et al. (2025)
Buzhong Yiqi Decoction	*Astragalus mongholicus* Bunge [Fabaceae], *Panax ginseng* C.A.Mey. [Araliaceae], *Atractylodes macrocephala* Koidz. [Asteraceae], *Angelica sinensis* (Oliv.) Diels [Apiaceae], *Citrus reticulata* Blanco [Rutaceae], *Actaea cimicifuga* L. [Ranunculaceae], *Bupleurum chinense* DC. [Apiaceae], *Glycyrrhiza glabra* L. [Fabaceae]	Quercetin, Astragaloside IV, licoisoflavanone, Stigmasterol	Anti-inflammatory action and immunomodulation	Hua et al. (2023), Zeng et al. (2025)
Shaoyao Decoction	*Paeonia lactiflora* Pall. [Paeoniaceae], *Rheum palmatum* L. [Polygonaceae], *Scutellaria baicalensis* Georgi [Lamiaceae], *Coptis chinensis* Franch. [Ranunculaceae]	Baicalin, Baicalein, Berberine, Palmatine, Paeoniflorin	Anti-inflammatory action, intestinal barrier repair, and regulation of apoptosis	Wang S. et al. (2024), Yang et al. (2025), Zhen et al. (2024), Wu et al. (2022)
Modified Baitouweng Decoction	*Pulsatilla chinensis* (Bunge) Regel [Ranunculaceae], *Coptis chinensis* Franch. [Ranunculaceae], *Phellodendron chinense* C.K.Schneid. [Rutaceae], *Fraxinus chinensis* Roxb. [Oleaceae], *Pleuropterus multiflorus* (Thunb.) Turcz. ex Nakai [Polygonaceae], *Glycyrrhiza glabra* L. [Fabaceae]	Quercetin, Isorhamnetin, Beta-sitosterol	Anti-inflammatory action, restoration of the intestinal barrier, and modulation of the gut microbiota	Wu et al. (2023a), Zhang et al. (2024), Qiu et al. (2025), Tingting et al. (2024), Deng et al. (2022), Gopika and Sachdeva (2025)
Xihuang Pill	*Calculus Bovis* , *Abelmoschus moschatus* Medik. [Malvaceae], *Boswellia sacra* Flück. [Burseraceae], *Commiphora myrrha* (T.Nees) Engl. [Burseraceae]	Acetyl-11-keto-β-boswellic acid, β-Boswellic acid, Bilirubin, Volatile oil	Anti-inflammatory action and promotion of mucosal repair	Qiu et al. (2025), Li et al. (2020b), Guo et al. (2015), Wang Z. et al. (2026), Xu and Li (2025)
Xuebijing Injection	*Carthamus tinctorius* L. [Asteraceae], *Paeonia lactiflora* Pall. [Paeoniaceae], *Conioselinum anthriscoides* ‘Chuanxiong’ [Apiaceae], *Salvia miltiorrhiza* Bunge [Lamiaceae], *Angelica sinensis* (Oliv.) Diels [Apiaceae]	Protocatechuic acid, Salvianolic acid C, Danshensu, Benzoyloxypaeoniflorin	Anti-inflammatory action, antioxidant action, and restoration of the intestinal barrier	Li et al. (2020a), Yuan et al. (2024)
TJ-14	*Pinellia ternata* (Thunb.) Makino [Araceae], *Coptis chinensis* Franch. [Ranunculaceae], *Scutellaria baicalensis* Georgi [Lamiaceae], *Zingiber officinale* Roscoe [Zingiberaceae], *Panax ginseng* C.A.Mey. [Araliaceae], *Ziziphus jujuba* Mill. [Rhamnaceae], *Glycyrrhiza glabra* L. [Fabaceae]	Baicalin, Glycyrrhizin, Berberine, Ginsenosides	Anti-inflammatory action and prophylaxis against chemotherapy-induced side effects	Murai et al. (2020), Miyashita et al. (2018), Arai et al. (2013), Yamada et al. (2013)

## Glossary

16S rRNA: 16S ribosomal RNA
ARE: Acute radiation enteritis
BTWD: Baitouweng decoction
BZYQD: Buzhong Yiqi decoction
CASP3: Caspase-3
CETSA: cellular thermal shift assay
CONSORT: Consolidated standards of reporting trials
COS: Core outcome set
CRP: C-reactive protein
DLS: dynamic light scattering
GQD: Gegenqinlian decoction
HIF-1: hypoxia-inducible factor 1
HV: Houhai acupoint
IL: interleukin
LGYD: Liangxue Guyuan Yishen decoction
mBTWD: Modified Baitouweng decoction
MyD88: Myeloid differentiation primary response 88
NF-κB: Nuclear factor kappa-light-chain-enhancer of activated B cells
Nrf2: Nuclear factor erythroid 2-related factor 2
PAINS: Pan-assay interference compounds
PD: Pharmacodynamic
PK: Pharmacokinetic
PK-PD: Pharmacokinetic-pharmacodynamic
PTGS1: prostaglandin-endoperoxide synthase 1
RCT: Randomized controlled trial
RE: Radiation enteropathy
ROS: Reactive oxygen species
SCFAs: Short-chain fatty acids
SPR: Surface plasmon resonance
STRICTA: Standards for reporting interventions in clinical trials of acupuncture
SYD: Shaoyao decoction/Shaoyao Gancao decoction
TCM: Traditional Chinese medicine
TGFβ: Transforming growth factor beta
TJ-14: Hangeshashinto
TLR4: Toll-like receptor 4
TNF-α: Tumor necrosis factor alpha
XBJ: Xuebijing injection
XHW: Xihuang pill
